# Evidence That Semaglutide Represents an Important Tool for Treatment of Irregular Menses and Chronic Anovulation in Women with Polyendocrine Metabolic Ovarian Syndrome

**DOI:** 10.3390/jcm15135165

**Published:** 2026-07-02

**Authors:** Enrico Carmina, Rosa Alba Longo

**Affiliations:** 1 School of Medicine, University of Palermo, 90139 Palermo, Italy; 2 Endocrinology Unit, FB Medical Center, 20121 Milano, Italy

**Keywords:** PMOS, PCOS, obese PMOS, chronic anovulation in PMOS, irregular menses, semaglutide, GLP-1 agonists, GLP-1 agonist treatment of PMOS

## Abstract

**Background:** Irregular menses and chronic anovulation are key components of Polyendocrine Metabolic Ovarian Syndrome (PMOS), but available treatments generally only mask the clinical problem, which presents itself again when the drugs are stopped. Because reduction of body weight in these patients is often associated with improvement of menstrual cycles, we evaluated the effects of treatment with semaglutide, a GLP-1 agonist that has emerged as an effective treatment for obesity. **Methods:** A total of 96 women with PMOS and body mass index (BMI) > 25 kg/m^2^ completed a six-month treatment protocol with semaglutide using an individualized dose-escalation regimen. Body weight, fasting glucose, insulin levels, insulin resistance (HOMA-IR), and ovulatory function were assessed before and after treatment. **Results:** After six months of treatment, mean body weight decreased significantly (−11.3 ± 5%, *p* < 0.01). Before treatment, 83% of PMOS patients presented with oligomenorrhea and anovulatory cycles. Following treatment, ovulatory cycles were observed in 52.5% of previously anovulatory women. The results were particularly good in overweight patients, with almost 95% of these PMOS patients achieving menstrual cycle normalization and ovulation, but also in patients with mild obesity. Results were less favorable in PMOS patients with moderate or severe obesity, but 25% of these patients achieved menstrual ovulatory cycle normalization when treated with semaglutide. **Conclusions:** This study represents an important therapeutic advancement, suggesting that women with PMOS and excessive body weight should be considered for treatment with GLP-1 receptor agonists before proceeding to therapies specifically aimed at inducing normal cycles and ovulation.

## 1. Introduction

Irregular menses and chronic anovulation are key components of Polyendocrine Metabolic Ovarian Syndrome (PMOS) [1,2,3,4] a common endocrine disorder [5] previously called Polycystic Ovary Syndrome (PCOS) [6]. Generally, PMOS patients with irregular menses are treated with estro-progestins, while the problem of chronic anovulation is addressed only when the fertility becomes an issue for the woman [4]. However, estro-progestins only mask the clinical problem, which presents itself again when the drugs are stopped.

Because excessive body weight and insulin resistance are found in most women with PMOS who with present irregular menses and chronic anovulation [2,3,4,5,7,8,9,10], lifestyle modification programs and/or metformin have been used to improve menstrual cyclicity and restore ovulatory cycles. In obese PMOS patients, lifestyle interventions have been shown to improve menstrual regularity and spontaneous fertility [4]. However, long-term adherence is often poor, and the resulting weight loss is frequently insufficient to restore ovulatory function [4,11,12,13,14]. Similarly, pharmacological therapies that improve insulin resistance, such as metformin, have shown only modest effects on body weight and irregular menses in women with PMOS [4,13,14].

More recently, glucagon-like peptide-1 (GLP-1) receptor agonists have emerged as effective treatments for obesity due to their ability to reduce appetite, delay gastric emptying, and improve glucose homeostasis [15,16]. Semaglutide, a long-acting GLP-1 receptor agonist, has shown substantial efficacy in promoting weight loss and improving glycemic control in obese patients with type 2 diabetes [16,17] and was initially marketed only for this population. However, this product is now marketed for the treatment of obesity independently of type 2 diabetes [17,18,19,20].

A few years ago, we published an initial study on the use of low doses of semaglutide in the treatment of obesity in women with PMOS who were unresponsive to a lifestyle program [21]. However, very few data on the effects of semaglutide on irregular menses and chronic anovulation in women with PMOS are available, particularly in large patient cohorts.

The aim of the present study was to evaluate the effects of semaglutide treatment on irregular menses and ovulatory function in a large group of women with PMOS and excessive body weight who were previously unresponsive to lifestyle intervention.

## 2. Materials and Methods

A total of 105 patients with PMOS and excess body weight (body mass index [BMI] > 25 kg/m^2^) were selected for possible six-month treatment with semaglutide. All patients had failed to respond to lifestyle modification programs. Because data on the safety of GLP-1 receptor agonists during pregnancy are lacking, contraception was recommended for all patients.

The diagnosis of PMOS was based on the Rotterdam criteria, fulfilling two out of three of the following criteria: menstrual irregularity suggestive of chronic anovulation, clinical or biochemical hyperandrogenism, and/or polycystic ovaries on ultrasound, after the exclusion of other medical disorders [1,22].

Semaglutide was administered using an individualized treatment protocol. Therapy started at 0.5 mg once a week by subcutaneous injection for one month, then was increased to 1 mg once weekly for the following two months. If body weight decreased by more than 5% from baseline during the first three months, treatment was continued at the same dose for another three months. If body weight decreased by less than 5% from baseline, the semaglutide dose was increased to 1.7 mg once weekly for the final three months.

Because cases of acute pancreatitis have been reported in patients treated with semaglutide [23], although this association has not been confirmed in large studies [17,24], pancreatic amylase levels were measured in all patients before treatment and every two months thereafter. In four patients, pancreatic amylase levels increased after two months of therapy, leading to treatment discontinuation. Amylase levels were then monitored monthly and returned to normal within two to three months. Semaglutide was not restarted in these cases.

Five additional patients withdrew from treatment with semaglutide because of severe gastrointestinal adverse effects, including vomiting and/or diarrhea.

Ninety-six PMOS patients completed the six-month semaglutide treatment protocol, and their data are reported in this study. This cohort was composed of three equal groups: overweight (*n* = 32, body mass index—BMI—25–29.9), mildly obese (*n* = 32, BMI 30–34.9), and moderately or severely obese (*n* = 32, BMI ≥ 35).

Menstrual cycle characteristics were assessed before and after six months of semaglutide treatment. Ovulation was assessed by measurement of serum estradiol and progesterone on days 21–22 of the menstrual cycle. Anovulation was defined as serum progesterone <3 ng/mL (<9.54 nmol/L). In patients with elevated serum estradiol levels but low serum progesterone levels, an additional progesterone measurement was performed one week later. In non-menstruating women, blood samples were obtained after withdrawal bleeding induced by progestogen administration. In patients with normal menstruation, serum progesterone was measured on days 21–22 of the cycle. In patients with normal menses, at least two consecutive menstrual cycles were studied, and a finding of low levels of serum progesterone (<3 ng/mL) in both cycles indicated the presence of chronic anovulation.

Fasting blood glucose and insulin levels were measured before and after six months of semaglutide treatment.

Serum insulin and progesterone were evaluated by previously reported methods [25]. Insulin sensitivity was calculated by the quantitative HOMA-IR method [26].

In all assays, intra-assay and inter-assay coefficients of variation did not exceed 6% and 15%, respectively.

No patient had received any medication for at least 3 months before the study, and all patients gave informed consent for this evaluation.

The research protocol obtained institutional approval from the Ethics Committee of the University of Palermo and FB Medical Center (2023–2024).

Statistical analyses were performed using Statview 5.0 (SAS Institute, Cary, NC, USA). Because several values were not normally distributed, a log transformation was necessary to obtain a normal distribution. Analysis of variance (ANOVA) followed by Tukey tests were performed to assess differences in changes in body weight and biochemical parameters between the different BMI subgroups of PMOS patients. A *p* < 0.05 was considered statistically significant. All results are reported as mean ± standard deviation (SD).

## 3. Results

Ninety-six PMOS patients (mean age 28.8 + 7.1, mean BMI 32.3 + 5.1, mean body weight 87.1 + 15.1 kg) completed treatment with semaglutide once a week for six months.

After three months of therapy with a low dose of semaglutide (0.5 mg once a week for one month followed by 1 mg once weekly for two months), 66 patients showed a reduction in body weight of greater than 5% and continued treatment with 1 mg once a week for an additional three months. The remaining 30 patients experienced a reduction in body weight of less than 5% and were treated with semaglutide at 1.7 mg once a week from month four to month six.

After six months of semaglutide treatment, mean body weight decreased significantly (*p* < 0.01) to 77 ± 15.6 kg (−10 + 4 kg: −11.3 + 5%) and mean BMI decreased to 28.6 + 5.5 (*p* < 0.01).

Most patients (*n* = 66, 68%) achieved a 10–25% reduction in body weight. However, 30 PMOS women (32%) lost less than 10% of their initial body weight. Of these, 12 were classified as partial responders (>5% but <10% weight loss), while 18 patients (19%) were non-responders to semaglutide treatment (<5% weight loss) (Table 1).

The greatest treatment effects were observed in overweight PMOS patients (*n* = 32; mean BMI, 26.6 ± 1; mean body weight, 71.5 ± 7 kg). Body weight normalized in 30 patients (92.5%), whereas 2 patients remained in the overweight range (Table 2). Among responders, mean weight loss was 11 ± 3 kg, corresponding to 15 ± 3% of initial body weight.

Good results were also observed in PMOS patients with mild obesity (*n* = 32, mean BMI 32.5 ± 1.6, mean body weight 87.9 ± 7.8 kg). Twenty patients (62.5%) showed a good response to treatment (>−10% of body weight loss, mean weight loss 12.5 ± 3 kg, and mean percentage weight loss 14 ± 3). In 18 patients (55%), body weight decreased to the overweight range, while in 2 patients (6.5%), body weight normalized (Table 2).

In PMOS patients with moderate or severe obesity (mean BMI 38 ± 2.6 and mean body weight 102 ± 11 kg), treatment effects were more varied. A total of 16 patients (50%) achieved a reduction in body weight greater than 10% (mean weight loss 15 ± 2 kg, and mean percentage weight loss 13 ± 6) (Table 2). However, no patient achieved normal body weight. BMI decreased to the overweight range in 4 PMOS patients (12.5%), while 18 patients (56%) moved to the mild obesity range (Table 2).

Before treatment, mean fasting blood glucose levels were in the normal range (92 ± 10 mg/100 mL), but 22 PMOS patients (23%) had increased fasting glucose (IFG, fasting blood glucose ≥100 <126 mg/dL). Mean serum insulin levels were slightly elevated (20 ± 12 μU/mL), and most patients (*n* = 70, 73%) had insulin resistance with a mean HOMA-IR value of 4.6 ± 3.

After treatment, fasting glucose, insulin, and HOMA-IR decreased significantly (*p* < 0.01). Sixteen of the twenty-two PMOS patients with previous IFG (73%) showed normalization of fasting glucose levels. Interestingly, normalization of fasting glucose also occurred in six patients who did not experience a decrease in body weight with semaglutide treatment.

Effect of semaglutide on ovulation and menstrual cyclicity.

Before treatment with semaglutide, 80 PMOS patients (83%) presented with oligomenorrhea and anovulatory cycles, whereas 16 PMOS patients had normal ovulatory cycles. After six months of treatment, 42 of the 80 anovulatory PMOS patients (52.5%) achieved a normalized menstrual cycle and developed ovulatory cycles.

Among overweight PMOS patients, 24 presented with oligomenorrhea and anovulatory cycles, while 8 had eumenorrhea and ovulatory cycles before treatment. After six months of semaglutide therapy, 22 of the 24 anovulatory PMOS patients achieved normalized menstrual cycles and developed ovulatory cycles (Table 3).

Among PMOS patients with mild obesity, 30 presented oligomenorrhea with anovulatory cycles, while 2 had eumenorrhea and ovulatory cycles. After treatment with semaglutide, 18 of the 30 anovulatory PMOS patients had eumenorrhea and ovulatory cycles (Table 3).

All 32 PMOS patients with moderate or severe obesity presented with oligomenorrhea and anovulatory cycles before treatment. After six months of therapy, eight of these PMOS patients developed eumenorrhea and ovulatory cycles (Table 3).

## 4. Discussion

Glucagon-like peptide-1 (GLP-1) receptor agonists are incretin analogs that are used to treat type 2 diabetes mellitus and obesity. These substances have several mechanisms of action, including reduction of gastric emptying, inhibition of glucagon secretion, beneficial changes in the intestinal microbiome, and direct effects on hypothalamic nuclei to enhance satiety (which promotes weight loss) [17,27]. Many other effects on the cardiovascular and nervous systems are emerging and will probably increase the use of these substances [27].

This study showed that semaglutide, a GLP-1 agonist, may represent an effective treatment for women with PMOS and excessive body weight who were unresponsive to lifestyle interventions. This therapy not only induced a significant reduction (>10%) in body weight but was also associated with normalization of menstrual cycles and restoration of ovulatory and potentially fertile ovarian cycles in 55% of previously anovulatory patients.

These results are particularly relevant for PMOS patients because they confirm that significant weight loss is often sufficient not only to reduce long-term metabolic and cardiovascular risks but also to normalize menstrual cycles and induce spontaneous fertility without the need for specific pharmacological treatments to induce ovulation [8]. This represents an important therapeutic advancement, suggesting that women with PMOS and excessive body weight should be considered for treatment with GLP-1 receptor agonists before proceeding to therapies specifically aimed at inducing ovulation.

Interestingly, these results were obtained using an individualized treatment protocol in which the dose of semaglutide was increased according to the degree of weight loss observed after an initial period of treatment with a low dose of semaglutide (0.5 mg once weekly for one month and 1.0 mg once weekly for two months). In patients who achieved a reduction in body weight of at least 5% within three months, the same dose (1.0 mg once weekly) of semaglutide was maintained for an additional three months, whereas for patients with a smaller weight reduction, the dose was increased to 1.7 mg once weekly from month four to month six of therapy.

The effects of treatment were particularly pronounced in patients with modest body weight excess (overweight), in whom approximately 90% achieved normalization of body weight and restoration of menstrual and ovulatory cycles. In PMOS patients with mild obesity, the results were also favorable, with approximately 60% of patients achieving a weight reduction greater than 10% and moving out of the obesity range, and 55% developing normal ovulatory cycles.

In contrast, the results were less favorable in patients with more severe obesity. Approximately 50% of these patients did not respond adequately to the treatment in terms of weight reduction. Nevertheless, approximately 25% of patients with moderate or severe obesity developed normal ovulatory cycles. This observation suggests that even moderate reductions in body weight, although insufficient to normalize BMI, may still improve ovarian function and allow the restoration of ovulatory cycles in some patients with PMOS.

The available data do not allow us to determine whether the observed reproductive effects of GLP-1 receptor agonists are mediated solely by weight reduction. Additional mechanisms, such as potential effects on insulin resistance and related metabolic pathways, may also contribute. Controlled studies are needed to distinguish between weight loss-dependent effects and potential direct effects on reproductive function.

One of the main advantages of our individualized treatment protocol was the relatively low prevalence of gastrointestinal side effects. Most patients did not report adverse effects, and among those who did, symptoms were generally mild and tended to decrease over time. Only a small proportion of patients (5%) discontinued the treatment because of severe gastrointestinal side effects.

Pancreatic amylase levels were also monitored during the treatment. In a small number of patients, treatment was discontinued because of an increase in pancreatic amylase levels to abnormally high values. Although recent large studies suggest that the risk of acute pancreatitis associated with GLP-1 receptor agonists is low [16,23], we chose a cautious approach and discontinued therapy in these cases.

In conclusion, in overweight and mildly obese PMOS patients, our findings suggest that semaglutide-associated weight loss is accompanied by improvement in menstrual cyclicity and ovulatory function. When administered using an individualized protocol in which the dose is increased according to the degree of weight loss, semaglutide frequently leads to normalization of body weight or at least to a reduction in BMI below the obesity threshold. These changes are associated with a marked reduction in metabolic risk and with restoration of ovulatory cycles in many previously anovulatory patients.

In PMOS patients with moderate or severe obesity, the effects of the treatment on body weight are generally not sufficient. However, in these women with PMOS, semaglutide treatment may provide clinically meaningful benefits, particularly the restoration of ovulatory cycles in a subset of patients.

## Figures and Tables

**Table 1:** Effect of semaglutide on body weight in 96 PMOS patients with excessive body weight.

Treatment	Mean Weight Loss (kg)	Mean Weight Loss (%)	Responders (>10%) *n* (%)	Partial Responders (5–10%) *n* (%)	Non-Responders (<5%) *n* (%)
Semaglutide (six months)	10 ± 4.7 **	11.4 ± 5.7 **	65 (68%)	12 (12%)	19 (20%)

Data are presented as mean ± SD or number (%). ** *p* < 0.01 versus baseline body weight.

**Table 2:** Effect of semaglutide on body weight in 96 PMOS patients divided according to their initial body weight.

	Responders (>10% Weight Loss) *n* (%)	Mean Weight Loss in Responders kg	Mean Weight Loss in Responders %	Normalization of Body Weight (BMI < 25) *n* %	Resolution of Obesity (BMI < 30) *n* %
Overweight *n* = 32	30 (92.5)	11 ± 3	15 ± 3	30 (92.5)	-
Mild obesity *n* = 32	20 (62.5)	12.5 ± 3	14 ± 3	2 (6.5%)	20 (62.5)
Moderate or severe obesity *n* = 32	16 (50)	15 ± 4	13 ± 6	0	4 (12.5)

Overweight: 25–29.9 kg/m^2^; Mild obesity: 30–34.9 kg/m^2^; Moderate/severe obesity: ≥35 kg/m^2^.

**Table 3:** Effect of semaglutide on ovulation in 96 PMOS patients divided according to baseline BMI.

	Ovulatory Patients Before Treatment *n* %	Ovulatory Patients After Treatment *n* %
Overweight *n* 32	8 (25)	30 (95)
Mild obesity *n* 32	2 (6)	20 (62.5)
Moderate/severe obesity *n* 32	0 (0)	8 (25)

BMI categories: Overweight: 25–29.9 kg/m^2^; Mild obesity: 30–34.9 kg/m^2^; Moderate/severe obesity: ≥35 kg/m^2^. Ovulation was assessed by serum progesterone >3 ng/mL in the mid-luteal phase.

## Data Availability

New data were created in this study and are available at FB Medical Center.

## References

[1] Rotterdam ESHRE-ASRM Sponsored PCOS Consensus Workshop Group (2004). Revised 2003 consensus on diagnostic criteria and long-term health risks related to polycystic ovary syndrome. Hum. Reprod..

[2] Guastella E., Longo R.A., Carmina E. (2010). Clinical and endocrine characteristics of the main PCOS phenotypes. Fertil. Steril..

[3] Yildiz B.O., Bozdag G., Yapici Z., Esinler I., Yarali H. (2012). Prevalence, phenotype and cardiometabolic risk of polycystic ovary syndrome under different diagnostic criteria. Hum. Reprod..

[4] Azziz R., Carmina E., Chen Z., Dunaif A., Laven J.S., Legro R.S., Lizneva D., Natterson-Horowtiz B., Teede H.J., Yildiz B.O. (2016). Polycystic ovary syndrome. Nat. Rev. Dis. Primers.

[5] Carmina E., Lobo R.A. (1999). Polycystic Ovary Syndrome (PCOS): Arguably the most common endocrinopathy is associated with significant morbidity in women. J. Clin. Endocrinol. Metab..

[6] Teede H.J., Khomami M.B., Morman R., Laven J.S., Joham A.E., Costello M.F., Patil M., Rees D.A., Berry L., Cree M.G. (2026). Polyendocrine metabolic ovarian syndrome, the new name for polycystic ovary syndrome: A multistep global consensus process. Lancet.

[7] Carmina E., Chu M.C., Longo R.A., Rini G.B., Lobo R.A. (2005). Phenotypic Variation in Hyperandrogenic Women Influences the Findings of Abnormal Metabolic and Cardiovascular Risk Parameters. J. Clin. Endocrinol. Metab..

[8] Shroff R., Syrop C.H., Davis W., Van Voorhis B.J., Dokras A. (2007). Risk of metabolic complications in the new PCOS phenotypes based on the Rotterdam criteria. Fertil. Steril..

[9] Carmina E., Nasrallah M.P., Guastella E., Lobo R.A. (2019). Characterization of metabolic changes in the phenotypes of women with polycystic ovary syndrome in a large Mediterranean population from Sicily. Clin. Endocrinol..

[10] Carmina E., Lobo R.A. (2022). Comparing Lean and Obese PCOS in Different PCOS Phenotypes: Evidence That the Body Weight Is More Important than the Rotterdam Phenotype in Influencing the Metabolic Status. Diagnostics.

[11] Wild R., Carmina E., Diamanti-Kandarakis E., Dokras A., Escobar-Morreale H., Futterweit W., Lobo R., Norman R.J., Talbott E., Dumesic D.A. (2010). Assessment of cardiovascular risk and prevention of cardiovascular disease in women with Polycystic Ovary Syndrome: A consensus statement of the Androgen Excess and PCOS (AE-PCOS) Society. J. Clin. Endocrinol. Metab..

[12] Welt C.K., Carmina E. (2013). The lifecycle of PCOS. J. Clin. Endocrinol. Metab..

[13] Naderpoor N., Shorakae S., de Courten B., Misso M.L., Moran L.J., Teede H. (2015). Metformin and lifestyle modification in polycystic ovary syndrome: Systematic review and meta-analysis. Hum. Reprod. Update.

[14] Apovian C.M., Aronne L.J., Bessesen D.H., McDonnell M.E., Murad M.H., Pagotto U., Ryan D.H., Still C.D. (2015). Pharmacological Management of Obesity: An Endocrine Society Clinical Practice Guideline. J. Clin. Endocrinol. Metab..

[15] Näslund E., Barkeling B., King N., Gutniak M., Blundell J.E., Holst J.J., Rössner S., Hellström P. (1999). Energy intake and appetite are suppressed by glucagon-like peptide-1 (GLP-1) in obese men. Int. J. Obes. Relat. Metab. Disord..

[16] Nauck M.A., Meier J.J. (2016). The incretin effect in healthy individuals and those with type 2 diabetes: Physiology, pathophysiology, and response to therapeutic interventions. Lancet Diabetes Endocrinol..

[17] Drucker D.J. (2024). Efficacy and Safety of GLP-1 Medicines for Type 2 Diabetes and Obesity. Diabetes Care.

[18] Singh G., Krauthamer M., Bjalme-Evans M. (2022). Wegovy (Semaglutide): A New Weight Loss Drug for Chronic Weight Management. J. Investig. Med..

[19] Phillips A., Clements J.N. (2022). Clinical review of subcutaneous semaglutide for obesity. J. Clin. Pharm. Ther..

[20] Müllertz A.L.O., Sandsdal R.M., Jensen S.B.K., Torekov S.S. (2024). Potent incretin-based therapy for obesity: A systematic review and meta-analysis of the efficacy of semaglutide and tirzepatide on body weight and waist circumference, and safety. Obes. Rev..

[21] Carmina E., Longo R.A. (2023). Semaglutide Treatment of Excessive Body Weight in Obese PCOS Patients Unresponsive to Lifestyle Programs. J. Clin. Med..

[22] Legro R.S., Arslanian S.A., Ehrmann D.A., Hoeger K.M., Murad M.H., Pasquali R., Welt C.K. (2013). Diagnosis and treatment of polycystic ovary syndrome: An Endocrine Society clinical practice guideline. J. Clin. Endocrinol. Metab..

[23] Singh S., Chang H.Y., Richards T.M., Weiner J.P., Clark J.M., Segal J.B. (2013). Glucagonlike peptide 1-based therapies and risk of hospitalization for acute pancreatitis in type 2 diabetes mellitus: A population-based matched case-control study. JAMA Intern. Med..

[24] Ayoub M., Chela H., Amin N., Hunter R., Anwar J., Tahan V., Daglilar E. (2025). Pancreatitis Risk Associated with GLP-1 Receptor Agonists, Considered as a Single Class, in a Comorbidity-Free Subgroup of Type 2 Diabetes Patients in the United States: A Propensity Score-Matched Analysis. J. Clin. Med..

[25] Carmina E. (1998). Prevalence of idiopathic hirsutism. Eur. J. Endocrinol..

[26] Carmina E., Stanczyk F.Z., Lobo R.A., Strauss J.F., Barbieri R.L., Dokras A., Williams C.J., Williams Z. (2023). Evaluation of hormonal status. Yen & Jaffe’s Reproductive Endocrinology.

[27] Rosen C.J., Ingelfinger J.R. (2026). GLP-1 Receptor Agonists. N. Engl. J. Med..

